# The association between basal metabolic rate and osteoarthritis: a Mendelian randomization study

**DOI:** 10.1186/s12920-023-01704-7

**Published:** 2023-10-24

**Authors:** Jingyu Zhou, Peng Wei, Feng Yi, Shilang Xiong, Min Liu, Hanrui Xi, Min Ouyang, Yayun Liu, Jingtang Li, Long Xiong

**Affiliations:** 1 Department of Orthopedics, The Second Affiliated Hospital of Nanchang University, No. 1 Minde Road, Nanchang, Jiangxi China; 2 The Second Clinical Medical College of Nanchang University, Nanchang, Jiangxi China; 3 Department of Traumatology, Jiangxi Provincial People’s Hospital, The First Affiliated Hospital of Nanchang Medical College, Nanchang, Jiangxi China; 4 Department of Orthopedics, The First Affiliated Hospital of Nanchang University, Nanchang, Jiangxi China

**Keywords:** Basal metabolic rate, Hip osteoarthritis, Knee osteoarthritis, Genome-wide association study, Mendelian randomization

## Abstract

**Background:**

The role of the basal metabolic rate (BMR) in osteoarthritis (OA) remains unclear, as previous retrospective studies have produced inconsistent results. Therefore, we performed a Mendelian randomization (MR) study to systematically investigate the causal relationship between the BMR and OA.

**Methods:**

Single-nucleotide polymorphism (SNP) data related to BMR and OA were collected in a genome-wide association study. Using OA as the outcome variable and BMR as the exposure factor, SNPs with strong correlation with the BMR as the tool variable were screened. The correlation between the BMR and OA risk was evaluated using the inverse-variance weighted method, and heterogeneity and pleiotropy were evaluated using a sensitivity analysis.

**Results:**

There was a potential causal relationship between the BMR and OA risk (odds ratio [OR], 1.014; 95% confidence interval [CI], 1.008–1.020; P = 2.29^e − 6^). A causal relationship was also revealed between the BMR and knee OA (OR, 1.876; 95% CI, 1.677–2.098; P = 2.98^e − 28^) and hip OA (OR, 1.475; 95% CI, 1.290–1.686; P = 1.26^e − 8^). Sensitivity analysis confirmed the robustness of these results.

**Conclusion:**

Here, we identified a latent causal relationship between the BMR and the risk of OA. These results suggest that the risk of OA in the hip or knee joint may be reduced by controlling the BMR.

**Supplementary Information:**

The online version contains supplementary material available at 10.1186/s12920-023-01704-7.

## Background

Osteoarthritis (OA), one of the most common degenerative diseases globally [1], is characterized by the degeneration of joint-related tissues (such as articular cartilage) and low-grade systemic inflammation, which eventually leads to joint instability and, in severe cases, physical disability [2]. OA significantly impacts the quality of life, life expectancy of affected individuals. In 2020, more than 500 million people worldwide had OA, accounting for 7% of the global population [3]. OA diagnosis and treatment places a great financial burden on society [4]; in Britain, OA is estimated to cost 1% of the gross national product. Underlying causes, such as trauma, obesity, or congenital abnormalities can directly or indirectly weaken cartilage and lead to chronic OA [5]. However, as the pathogenesis of OA remains unclear, there are currently no effective methods for preventing this chronic disease.

Some studies have shown that work-related physical activity can significantly increase the risk of knee OA [6], and long-term exposure to high-intensity physical exercise has been associated with knee and hip OA [7]. Cross-sectional studies have shown the basal metabolic rate (BMR), which is the energy needed for basic physiological activities when people are awake, calm, and unaffected by other factors [8], is 5–20% higher in individuals who have undergone exercise training than in sedentary individuals [9, 10], suggesting a possible relationship between the BMR and OA [11, 12]. Therefore, the relationship between the BMR and OA remains unclear.

Previous studies on OA have reported that cellular metabolism can influence its progression [13, 14]. However, these studies mainly focused on mechanistic research, and their conclusions are unclear. Traditional observational epidemiological studies have limited sample sizes and may be biased by confounding factors and reverse causal correlations. Randomized controlled trials, the gold standard for determining clear causal relationships, can reduce selection bias and confounding factors. However, these trials are challenging to implement and involve considerable personnel and resources. Moreover, because of their strict inclusion and exclusion criteria, the conclusion may lack generalisability. Mendelian randomization (MR), as the ‘randomized controlled trials’ of nature, minimizes the limitations of observational studies by using genetic variation as an instrumental variable to infer potential causal relationships between genetically influenced exposures and outcomes [15]. Therefore, this study used MR to evaluate the potential causal effects of BMR on OA, using the BMR as an exposure and OA as an outcome.

## Materials and methods

### Overview of project design

**Fig. 1:**
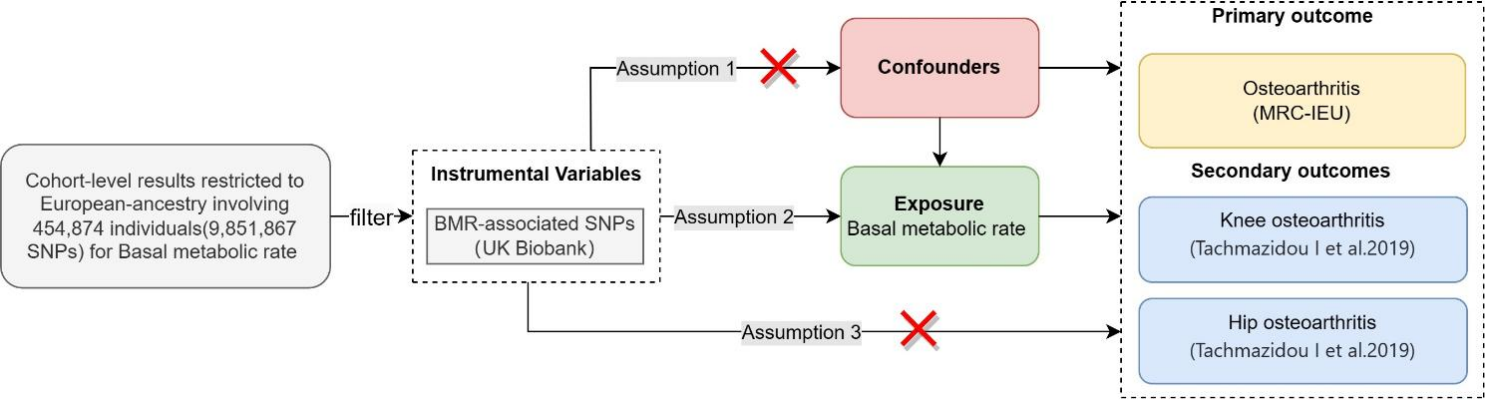
The schematic representation of this study and the three assumptions of Mendelian randomization analysis: (1) The used genetic IVs are independent of the currently known confounding factors. (2) The selected IVs are closely related to BMR. (3) The selected SNPs have no direct influence on the outcome of osteoarthritis, but only influence the outcome through basal metabolic rate.

An overview of the MR study design is shown in Fig. 1. The MR analysis was based on three assumptions [16]: (1) The selected genetic IVs (Instrumental variables) are devoid of known confounding factors. (2) The selected IVs is closely related to the BMR. (3) The selected single-nucleotide polymorphisms (SNPs) had no direct influence on the outcome of OA but only influenced OA indirectly through the BMR. The MR study outcomes included primary and secondary outcomes; the primary outcome was OA and the secondary outcomes were site-specific knee and hip OA.

### Genetic associations with outcomes

The data analysed in this study was obtained from the Medical Research Council Integrated Epidemiology Unit open genome-wide association study (GWAS; http://gwas-api.mrcieu.ac.uk/, accessed on 27 December, 2022). Primary outcome data was obtained from an OA dataset (GWAS ID: ukb-b-14486) including 462,933 individuals from Europe (424,461 healthy controls and 38,472 patients with OA), with a total of 9,851,867 SNPs. Site-specific secondary outcome data was obtained from two datasets (GWAS ID: ebi-a-GCST007090; GWAS ID: ebi-a-GCST007091) of the same GWAS, a meta-analysis of the whole OA genome using UK Biobank and arcOGEN resources [17]. Dataset ebi-a-GCST007090 included 378,169 healthy controls and 24,955 patients with knee OA and a total of 29,999,696 SNPs; the ebi-a-GCST007091 dataset included 378,169 healthy controls and 15,704 patients with hip OA and a total of 29,771,219 SNPs. Table 1 summarizes the GWAS data used in this study.

**Table 1:** Summary of the GWAS included in this MR study

Exposure/Outcomes	Dataset	Sample size	Number of SNPs	Population	Consortium	Sex	Year
Basal Metabolic Rate	ukb-b-16446	454,874	9,851,867	European	MRC-IEU	Males and Females	2018
Primary Outcome (Osteoarthritis)	ukb-b-14486	462,933	9,851,867	European	MRC-IEU	Males and Females	2018
Secondary Outcome (Knee Osteoarthritis)	ebi-a-GCST007090	403,124	29,999,696	European	Tachmazidou I	NA	2019
Secondary Outcome (Hip Osteoarthritis)	ebi-a-GCST007091	393,873	29,771,219	European	Tachmazidou I	NA	2019

### Selection of instrumental variables

To satisfy assumption 2, we ensured that the selected SNPs were strongly correlated with the BMR. SNPs with *P* < 5^e − 8^, genetic distance 10,000 kb, and r^2^ < 0.001 were identified in the ukb-b-16,446 dataset using the “clump_data” function in the “TwoSampleMR” package of the RStudio application version 4.2.1. To satisfy assumption 1, the confounding factors such as body mass index, inflammatory indicators, cholesterol, bone mineral density, and smoking were identified in the datasets using the PhenoScanner database (http://www.phenoscanner.medschl.cam.ac.uk/ accessed on 1 January, 2023). A total of 73 SNPs associated confounding factors (Additional file 1: Table S1) were excluded. Finally, the included data were evaluated using an MR pleiotropy residual sum and outlier (MR-PRESSO) test in the “MR-PRESSO” package of the RStudio application version 4.2.1 to remove potential outliers (Additional file 1: Table S2). The remaining SNPs were included in the subsequent analysis.

R^2^ represents the proportion of variation in the BMR explained by SNPs [18] and denotes the degree of exposure of the IV interpretation; as shown in Formula 1:

1 $${R}^{2}=2\times EAF\times \left(1-EAF\right)\times {\beta }^{2}$$

where EAF represents the minor allele frequency and β represents the effect of SNPs on exposure.

To eliminate bias caused by instrumental variables, we calculated F-statistic. SNPs with an F-statistic < 10 were considered weak instruments and excluded from the MR analysis [19]. F-statistic was calculated using Formula 2:

2 $$F=\frac{N-K-1}{K}\times \frac{{R}^{2}}{1-{R}^{2}}$$

where N represents the exposed GWAS sample size and K represents the number of IV.

### Statistical analysis and sensitivity analysis

In this MR study, we used the IVW method, weighted median (WME) method, MR-Egger method, weighted model, and simple model to evaluate the causal relationship between the BMR and the risk of OA. The IVW method does not consider the existence of an intercept in the regression and uses the reciprocal of the outcome variance as the weight for fitting [20]. A fixed- or random-effects model was selected for the IVW test based on heterogeneity [21]. Unlike the IVW method, the MR-Egger method considers the existence of an intercept and uses the reciprocal of the variance of the tool variables as the weight-to-fit. Considering the Instrument Strength Independent of Direct Effect assumption, the results of the MR-Egger method are valid even if SNPs have pleiotropy [22]. The WME method was used to sort the estimated values of individual SNPs according to their weights. When at least 50% of the SNPs are effective instrumental variables, the causal effects of exposure and outcome can be determined using this method [23, 24]. The weighted method evaluates causal effects according to clusters containing a large number of SNPs. As the IVW method usually has a significantly higher statistical efficacy than other those of the MR methods, it was used as the main method of identifying potential causality [25]. Results are presented as odds ratios (OR) and 95% confidence intervals (CI).

We conducted a sensitivity analysis to evaluate the robustness of the MR results. We calculated the P-value of the MR-Egger regression intercept to determine whether SNPs were pleiotropic. MR-Egger regression can test for pleiotropic bias and is a tool for detecting research bias in meta-analyses [26]. Cochran’s Q test was used to evaluate the heterogeneity of the effect size estimation for single SNPs. To alleviate potential associations between outcomes and exposure, we used the Steiger directionality test to identify the correct causal direction [27]. Finally, the robustness of the MR results was assessed by excluding each IV individually to prevent a single SNP from significantly interfering with the final result. The directional horizontal pleiotropy of the IV was visually displayed through funnel plots using a single Wald ratio for each SNP.

The occurrence of false positives was reduced using the Bonferroni correction (P = 0.05/N, where N is the number of MR methods used). All analyses were performed using the “TwoSampleMR” and “MR-PRESSO” packages of the RStudio application.

## Results

### Primary MR analysis: influence of the BMR on OA

The F-statistics of the individual SNPs included in the analysis ranged from 11.7686 to 171.7862, and the sum of R^2^ was 2.5781%, indicating that there was no weak instrumental variable in this analysis. There was significant heterogeneity in Cochran’s Q test (Q = 600.5889, P = 7.93^e − 8^); therefore, we used a multiplicative random-effects model to ensure the robustness of the MR analysis results. Three outliers were identified by the MR-PRESSO test (Additional file 1: Table S2), which were removed and analyzed using MR (Additional file 1: Table S3). Our IVW-MR approach revealed that an increased BMR was significantly associated with an increased risk of OA (OR, 1.014; 95% CI, 1.008–1.020; P = 2.29^e − 6^). Similar results were obtained using the WME analysis (OR, 1.015; 95% CI, 1.007–1.024; P = 5.27^e − 4^). There was no statistically significant difference in the MR-Egger analysis (OR, 1.009; 95% CI, 0.993–1.025; P = 2.59^e − 1^). The removing a certain SNP using the leave-one-out method (Additional file 2: Figure S1). The MR pleiotropy test showed no horizontal pleiotropy by the MR-Egger regression (intercept = 7.036^e − 5^, se = 1.041^e − 4^, P = 0.499). The Steiger directionality test found the results of the analysis to be consistent with the expected direction, and this direction did not change following outlier removal (P = 0.922).

### Secondary MR analysis: influence of BMR on knee OA

The F-statistics of the individual SNPs included in the analysis were 10.2481–149.5920 and the sum of R^2^ was 2.5754%, indicating that there was no weak instrumental variable in this analysis. There was significant heterogeneity in Cochran’s Q test (Q = 831.2233, P = 1.06^e − 27^); therefore, we used a multiplicative random-effects model to ensure the robustness of the MR analysis results. Two outliers were identified by the MR-PRESSO test (Additional file 1: Table S2), which were removed and analyzed using MR (Additional file 1: Table S4). Our IVW-MR approach revealed that an increased BMR was significantly associated with an increased risk of knee OA (OR, 1.876; 95% CI, 1.677–2.098; P = 2.98^e − 28^). This result was also reflected in the WME analysis (OR, 1.836; 95% CI, 1.604–2.102; P = 1.33^e − 18^). There was no statistically significant difference in the MR-Egger analysis (OR, 1.427; 95% CI, 1.059–1.923; P = 2.01^e − 2^). The removal of SNPs using the leave-one-out method had no significant influence on the results (Additional file 2: Figure S1). No horizontal pleiotropy was identified by the MR-Egger regression (intercept = 3.798^e − 3^, SE = 1.960^e − 3^, P = 0.053). The Steiger directionality test found the results of the analysis to be consistent with the expected direction, and this direction did not change following outlier removal(*P* = 0.712).

### Secondary MR analysis: influence of BMR on hip OA

The F-statistics of the individual SNPs included in the analysis were 10.0129–146.1592 and the sum of R^2^ was 2.5267%, indicating that there was no weak instrumental variable in this analysis. There was significant heterogeneity in Cochran’s Q test (Q = 727.6431, P = 2.32^e − 18^); therefore, we used a multiplicative random-effect models to ensure the robustness of the MR analysis results. Nine outliers were identified by the MR-PRESSO test (Additional file 1: Table S2), which were removed and analyzed using MR (Additional file 1: Table S5). Our IVW-MR approach revealed that an increased BMR was significantly associated with an increased risk of hip OA (OR, 1.475; 95% CI, 1.290–1.686; P = 1.26^e − 8^). The same conclusion was reached using the WME method (OR, 1.299; 95% CI, 1.092–1.546; P = 3.17^e − 3^). There was no statistically significant difference in the MR-Egger analysis (OR, 1.147; 95% CI, 0.800–1.644; P = 4.56^e − 1^). The removal of SNPs using the leave-one-out method had no significant influence on the results (Additional file 2: Figure S1). No horizontal pleiotropy was identified by the MR-Egger regression (intercept = 3.481^e − 3^, SE = 2.359^e − 3^, P = 0.141). The Steiger directionality test found the results of the analysis to be consistent with the expected direction, and this direction did not change following outlier removal *(P* = 0.446).

**Fig. 2:**
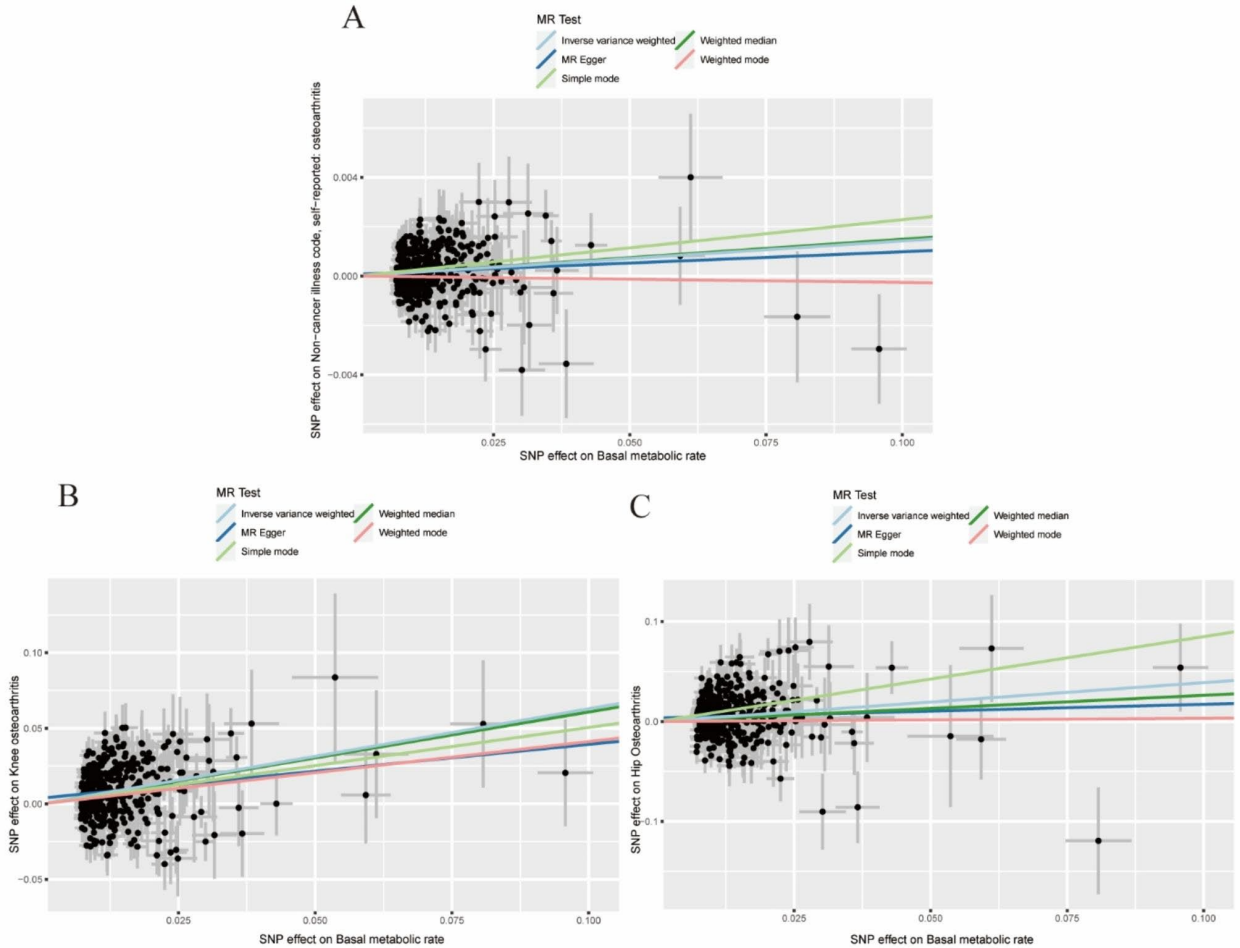
Scatter plots of causality. The slope of each line corresponding to the estimated MR effect in different models. (**A**) Primary outcome (Osteoarthritis); (**B**) Secondary outcome (Knee Osteoarthritis); (**C**) Secondary outcome (Hip Osteoarthritis)

**Fig. 3:**
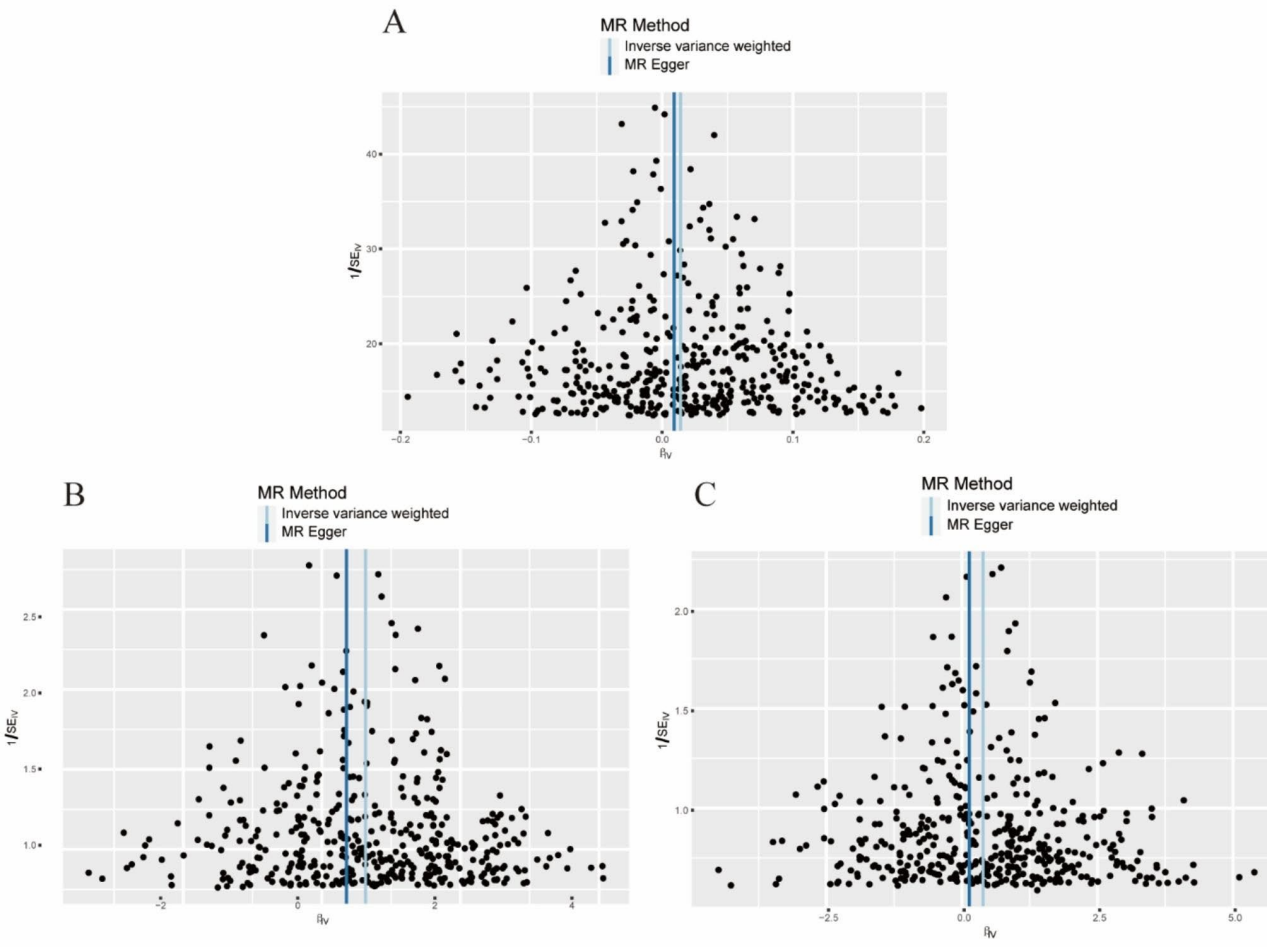
Funnel plot of causality. (**A**) Primary outcome (Osteoarthritis); (**B**) Secondary outcome (Knee Osteoarthritis); (**C**) Secondary outcome (Hip Osteoarthritis)

**Fig. 4:**
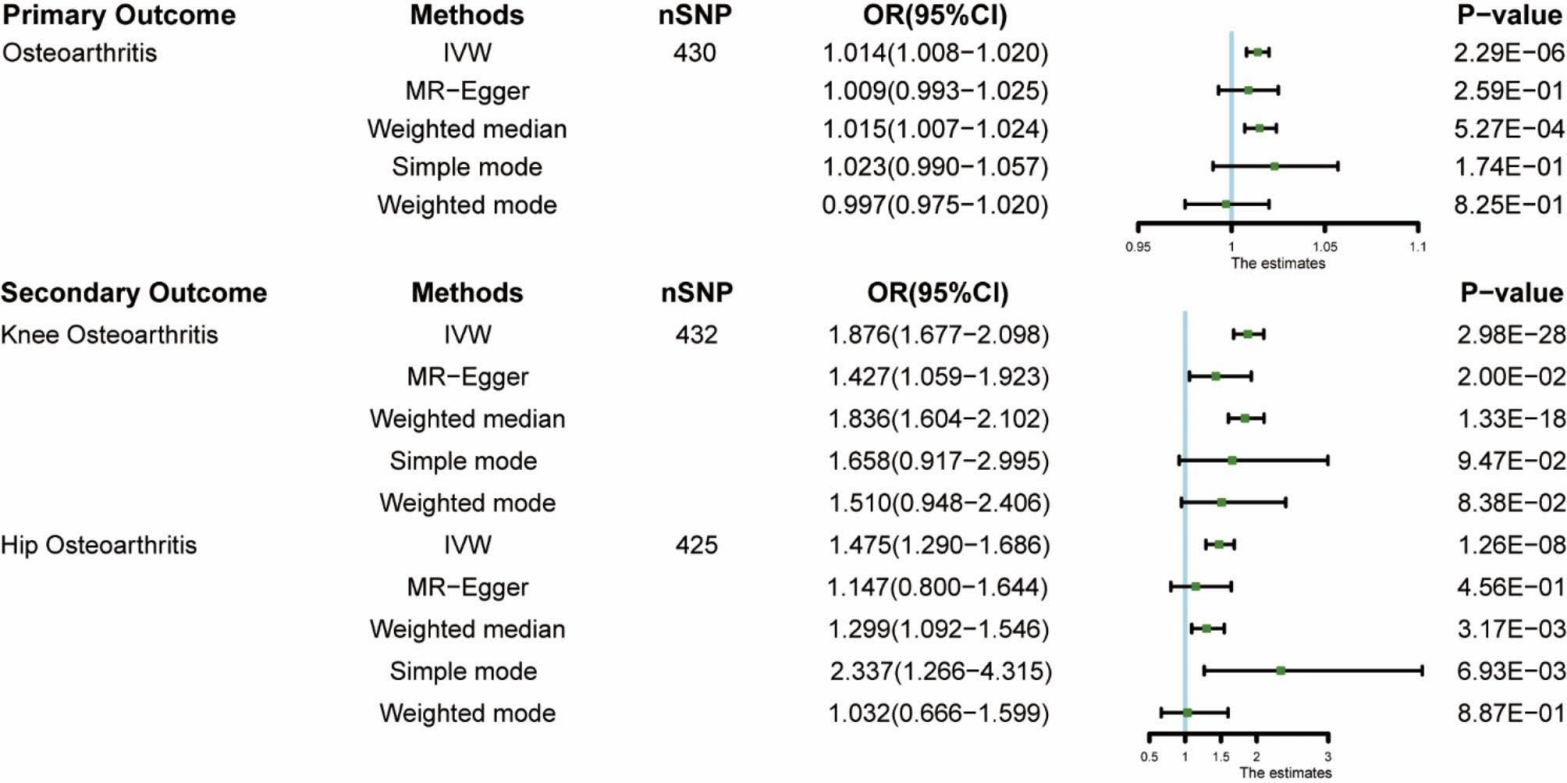
Causal estimates given as odds ratios and 95% confidence intervals for the effect of basal metabolic rate on osteoarthritis by different consortium

The forest plot (Additional file 2: Figure S2) shows the MR effect distribution of each IV in the IVW model. The scatter plot (Fig. 2) shows the effects of the different MR methods, and the funnel plots (Fig. 3) of each group are approximately symmetrical; therefore the results are unlikely to be affected by potential deviations. Figure 4 shows the causal effect estimation of the relationship between BMR and OA by different MR methods. Table 2 presents the details of the sensitivity analysis.

**Table 2:** Sensitivity analysis of primary and secondary MR analyses

Outcome	Number of IVs	R^2^	F	Heterogeneity test		MR-Egger pleiotropy test		Steiger directionality test		MR-PRESSO Distortion Test
				Q	*p*-Value	Intercept	*p*-Value	Causal direction	*p*-Value	*p*-Value
Osteoarthritis	430	2.5781%	28.4632	600.5889	7.93 e-08	7.036 e-05	0.499	TRUE	< 0.0001	0.922
Knee Osteoarthritis	432	2.5754%	24.6410	831.2233	1.06 e-27	3.798 e-03	0.053	TRUE	< 0.0001	0.712
Hip Osteoarthritis	425	2.5267%	23.9976	727.6431	2.32 e-18	3.481 e-03	0.141	TRUE	< 0.0001	0.446

## Discussion

Our MR study reveals causal relationships between the BMR and OA (OR, 1.014; 95% CI, 1.008–1.020), This result was also observed in OA of the knee (OR, 1.876; 95% CI, 1.677–2.098) and OA of the hip (OR, 1.475; 95% CI, 1.475–1.686).

The BMR plays a significant role in maintaining bodily functions. As such, an increase in the BMR can indicate metabolic or endocrine system disorders, and the BMR has been shown to be associated with many metabolic diseases, such as cancer and osteoporosis [28, 29]. Diseases characterized by an immune response have also been associated with an increased BMR due to a bioenergy imbalance [30].

OA, a chronic inflammatory and metabolic disease, is often accompanied by synovial hyperplasia and low-grade inflammatory infiltration of the synovial intima. Several biological mechanisms can explain the positive correlation between a high BMR and OA risk. Individuals with a high BMR require more cellular energy to meet their metabolic needs [29]. Mitochondria are the main source of cellular metabolism and the mitochondrial respiratory chain is a primary source of cellular reactive oxygen species. Reactive oxygen species are considered a potential cause of OA due to their damaging effects on DNA [31], which can result in synovitis and subchondral bone dysfunction [32]. In addition, metabolism is associated with chronic inflammation [33]. At a high BMR, inflammation and degradation of protein biosynthesis are increased, leading to catabolism and the progression of OA [34]. Excessive metabolite and nutrient production can also cause inflammation [35]. Low-grade inflammation plays a key role in the pathogenesis of OA [36], through effects on the differentiation and function of chondrocytes and the expression of metalloproteases and aggregates, which result in cartilage degradation and joint degeneration [37]. Perisynovial macrophages have been shown to be essential for the formation of peri-cartilaginous osteophytes [38].

However, previous retrospective studies have not resulted in a consensus on whether the BMR has a direct effect on OA. Muscle metabolism is an important part of the BMR, as shown by the positive correlation between skeletal muscle mass index and the BMR (r = 0.72, β = 30.96, P < 0.01) [39]. A retrospective study showed that the skeletal muscle mass index (and the BMR) decreases with age, which may be related to age-related OA development and progression [39]. OA is a complex process affected by many factors, such as age, sex, and joint wear and tear during exercise. These may act as confounding factors in the retrospective analysis of OA risk. Individuals who undertake regular exercise may experience joint wear and tear or trauma, which may lead to OA [40], independent of the effects of muscle metabolism. In addition, after being affected by physical activity, there was no significant difference in the risk of OA among different genders [41, 42]. Aging is associated with a decrease in cell proliferation and tissue regeneration, and subsequent functional impairment [43]. Aging chondrocytes accumulate in the articular cartilage, further promoting OA development [44].

This study is the first to evaluate the causal relationship between the BMR and OA at the genetic level. using three GWAS datasets related to OA and five different models to evaluate causality. A strong causal relationship between BMR and OA (including knee and hip OA) was observed. This suggests that individuals with a high BMR should be aware of the associated risk of OA in knee and hip joints. This study introduced the concept of instrumental variables to explore the potential causal relationship between BMR and OA from a genetic perspective. This indicates the significance of BMR in OA and provides a novel idea for the future research on the prevention of OA.

### Limitation

Despite our best efforts, our study has some limitations. First, the GWAS database included data obtained from a European population; therefore, it is unknown whether our conclusions are applicable to non-European populations. Second, we analyzed OA at different sites as the secondary outcome, and owing to database limitations, we were unable to evaluate the sample population for subgroup stratification, such as by sex, age, and region, to explore the robustness of this conclusion in different subgroups. In addition, patients self-reported OA, which raises the possibility of misdiagnosis. Third, the association between the BMR and OA risk can only be preliminarily confirmed using the MR method, and the potential biological mechanism underlying this association remain unclear. Therefore, further basic research is required to investigate in detail the relationship between these factors needs further and more detailed basic research to confirm. Finally, the causal results provided by certain analysis methods, such as MR-egger, were inconsistent with those of the main analysis using the IVW method, indicating the existence of pleiotropy. Although no obvious horizontal pleiotropy was found in the sensitivity analysis, it was difficult to verify the hypothesis that genetic tools can only influence the results through exposure factors, instead of vertical pleiotropy. In addition, it is difficult to completely rule out pleiotropy because the functional biological effects of these SNPs are not yet fully understood.

## Conclusion

In conclusion, this study identifies BMR as a causal risk factor for OA in both knee and hip joints, which means that individuals with high BMR levels need to pay more attention to the risk of osteoarthritis in the knee and hip joints.

### Electronic supplementary material

Below is the link to the electronic supplementary material.

Supplementary Material 1

Supplementary Material 2

## Data Availability

The data that support the findings of this study are available from the corresponding author upon reasonable request. The datasets generated for this study can be found in the IEU OpenGWAS project (https://gwas.mrcieu.ac.uk/datasets/ukb-b-16446/, https://gwas.mrcieu.ac.uk/datasets/ukb-b-14486/, https://gwas.mrcieu.ac.uk/datasets/ebi-a-GCST007090/, https://gwas.mrcieu.ac.uk/datasets/ebi-a-GCST007091/ ).

## Abbreviations

BMR: Basal metabolic rate
CI: Confidence interval
GWAS: Genome-wide association study
IV: Instrumental variable
IVW: Inverse-variance weighted
MR: Mendelian randomization
MR-PRESSO: Mendelian randomization pleiotropy residual sum and outlier
OA: Osteoarthritis
OR: Odds ratio
SNP: Single-nucleotide polymorphism
WME: Weighted median
