# Supplementary material for: The association between basal metabolic rate and osteoarthritis: a Mendelian randomization study
Source: BMC Med Genomics. 2023 Oct 24;16:258. doi: 10.1186/s12920-023-01704-7 (PMC10594886; doi:10.1186/s12920-023-01704-7)
Supplement: Supplementary file 2 — Supplementary Material 2 [file 12920_2023_1704_MOESM2_ESM.docx]

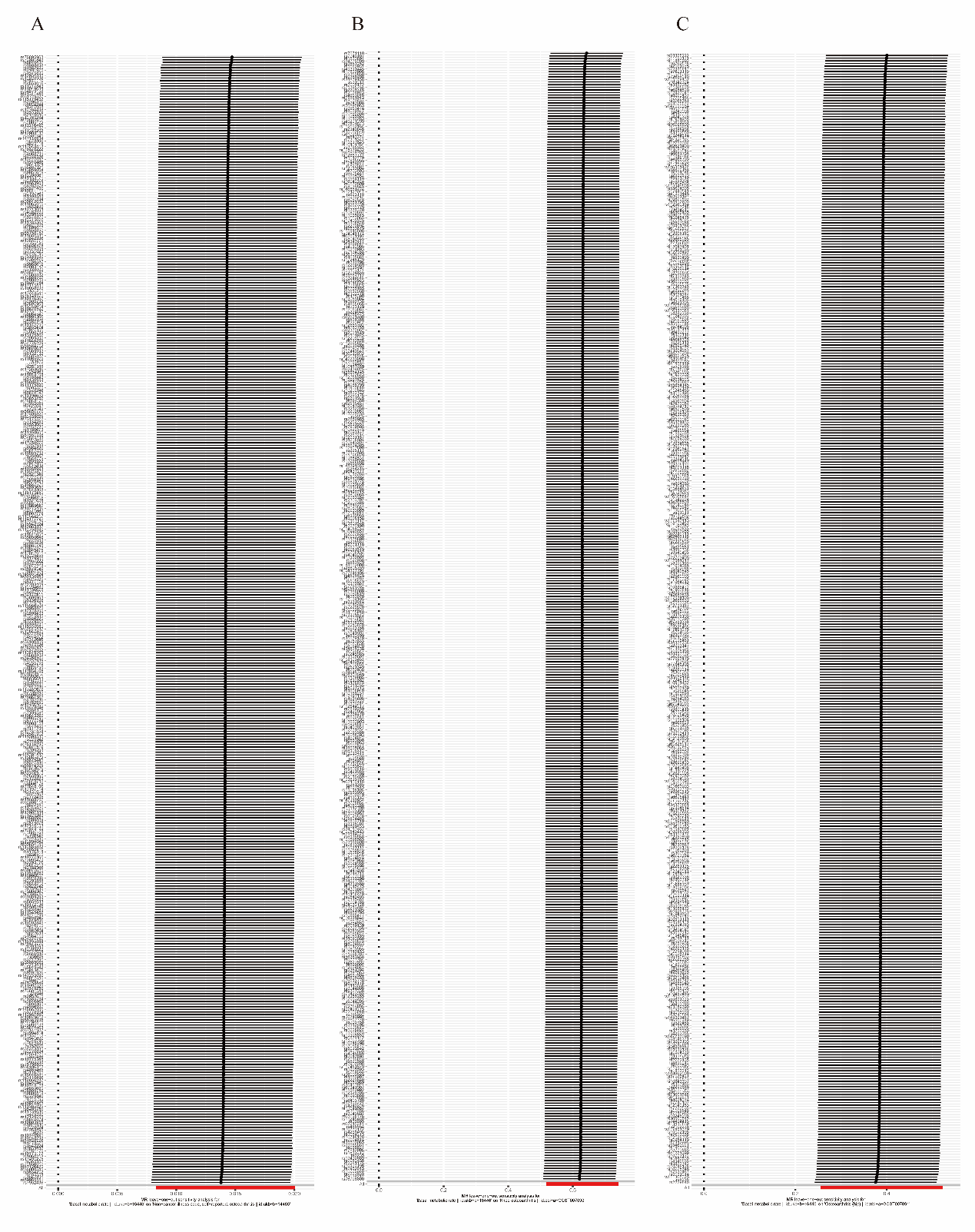
Figure S1. Leave one out of sensitivity tests. (A) Primary outcome (Osteoarthritis); (B) Secondary outcome (Knee Osteoarthritis); (C) Secondary outcome (Hip Osteoarthritis).


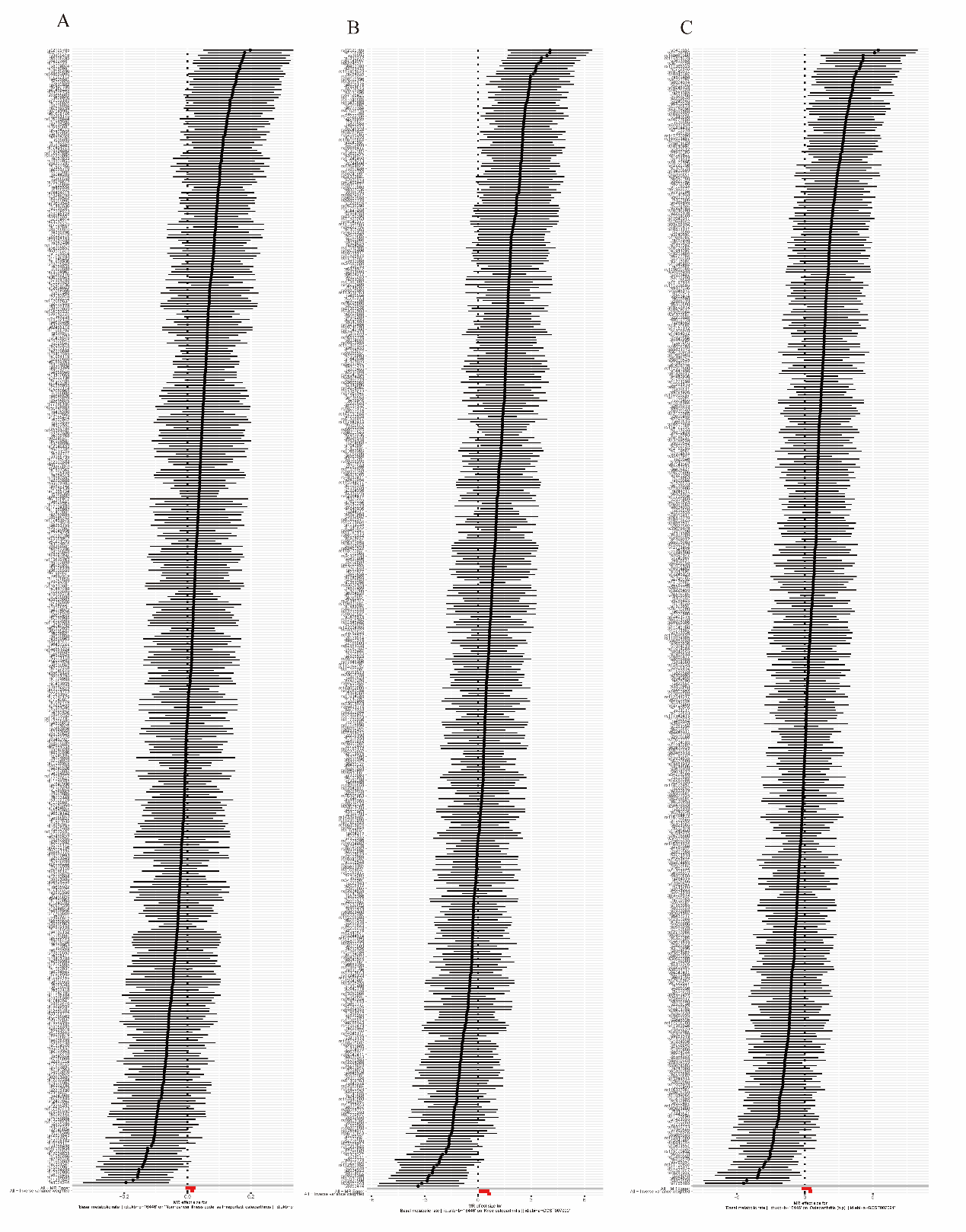
Figure S2. Detailed forest plots with the estimated MR effect of each IV in IVW models (A) Primary outcome (Osteoarthritis); (B) Secondary outcome (Knee Osteoarthritis); (C) Secondary outcome (Hip Osteoarthritis).
